# Inhibition of connective tissue growth factor by small interfering ribonucleic acid prevents increase in extracellular matrix molecules in a rodent model of diabetic retinopathy

**Published:** 2012-04-07

**Authors:** Jennifer L. Winkler, Mamdouh H. Kedees, Yelena Guz, Gladys Teitelman

**Affiliations:** State University of New York, Downstate Medical Center, Department of Cell Biology, Brooklyn, NY

## Abstract

**Purpose:**

Connective tissue growth factor (CTGF) is a profibrotic factor that induces extracellular matrix (ECM) production and angiogenesis, two processes involved in diabetic retinopathy (DR). In this study, we examined whether insulin therapy or a *CTGF*-specific small interfering RNA (siRNA) administered to diabetic rats decreased the levels of CTGF and of selected putative downstream genes in the retina.

**Methods:**

Rats with streptozotocin-induced diabetes were used. Animals received either no treatment for 12 weeks or were administered constant insulin therapy. MRNA and protein levels of *CTGF* and select ECM genes were determined using real-time PCR and western blotting of the retina. Localization of CTGF in the retina was visualized using immunohistochemistry. A group of diabetic rats received intravitreal injection of *CTGF* siRNA, and the retinas were examined three days later.

**Results:**

CTGF mRNA and protein significantly increased in the retinas of diabetic rats. Immunohistochemistry indicated that retinal Müller cells of diabetic rats expressed CTGF. Hyperglycemia upregulated mRNA levels of fibronectin, laminin β1, collagen IVα3, and vascular endothelial growth factor (VEGF), and this increase was prevented by insulin therapy. Treatment of diabetic rats with CTGF siRNA decreased laminin β1, collagen IVα3 mRNA, and CTGF mRNA and protein but did not affect fibronectin or vascular endothelial growth factor mRNA levels.

**Conclusions:**

These results indicate that *CTGF* and ECM genes can be regulated using insulin. Importantly, these results also suggest that CTGF regulates changes in ECM molecules in DR.

## Introduction

Diabetic retinopathy (DR) is the leading cause of visual impairment and blindness among adults of working age in the United States [1]. DR can be divided into two stages. The first stage is non-proliferative DR, characterized by retinal edema, microaneurysms, venous bleeding, and soft exudates. The second stage, proliferative DR, is characterized by angiogenesis, retinal detachment, blindness, and an increased number of blood vessels with altered vascular permeability. DR occurs because of altered blood flow, pericyte loss, tissue hypoxia, and basement membrane thickening provoked by increased production of collagen IV, laminin, and fibronectin [2-4]. These changes were detected after 12 and 17 weeks following the appearance of diabetes, respectively [5,6]. In addition, there is also dysregulation of remodeling proteins such as matrix metalloproteinease-2, matrix metalloproteinease-9 (MMP-9), plasminogen activator inhibitor-1, tissue inhibitor of metalloproteinease-1, and other proteins [7-9].

Connective tissue growth factor (CTGF) is a profibrotic factor that induces extracellular matrix (ECM) production and angiogenesis [10], two processes involved in the development of DR. CTGF is one of the six members of the CCN family of proteins. The CCN acronym is derived from the names of the first three members of the family of proteins: Cyr61 (cysteine-rich protein 61), CTGF, and NOV1 (nephroblastoma overexpressed gene-1). The CCN family of proteins is involved in a wide range of functional pathways such as cell adhesion, cell survival, angiogenesis, tumorigenesis, and wound healing [11]. *CTGF* is upregulated in human and rodent models of DR [12,13] and is induced by glucose [5,13] and advanced glycation end-products [5]. In addition, *CTGF* is upregulated by vascular endothelial growth factor (VEGF) [14,15], which is increased in patients with diabetes and is a critical regulator of vascular permeability and angiogenesis [16].

The exact role of CTGF in the progression of DR has yet to be determined. Although *CTGF* knockout is embryonic lethal [17], *CTGF* heterozygote mice have a 50% decrease in CTGF levels in plasma and urine and show decreased retinal basal lamina thickening in diabetes [6]. In addition, CTGF is responsible for the development of fibrosis, not angiogenesis, which results in scarring of the retina and blindness [18]. Studies of the kidney strengthened the possibility that CTGF mediates the alterations of ECM during hyperglycemia [19].

In this study, we sought to determine the role of CTGF in non-proliferative DR. First, we tested whether the increase in CTGF levels with hyperglycemia could be attenuated through insulin therapy and whether this treatment affected the level of expression of key ECM molecules. Since glycemic levels fluctuate during insulin therapy, we also tested whether a specific inhibition of *CTGF* using siRNA affects the levels of selected ECM molecules that increase in the diabetic retina.

## Methods

### Diabetic animal model

Male Sprague Dawley Rats (Charles River, Troy, NY), weighing approximately 200 g, received a single (IP) injection of 80 mg/kg streptozotocin (STZ; Sigma, St. Louis, MO) dissolved in 0.1 M citrate buffer (pH 4.5) [20]. Control non-diabetic animals were injected with an equal volume of citrate buffer. Fasting blood glucose (FBG) levels were measured using a PrecisionXtra blood glucose monitor (Abbot, Alameda, CA). Animals with FBG greater than 350 mg/dl were considered diabetic. The first day of recorded hyperglycemia was considered day 1 of the experiment. Animals were euthanized with Euthasol (120 mg/kg; Vibrac Corp., Fort Worth, TX) and sacrificed after 8 and 12 weeks of hyperglycemia. Eyes were enucleated, and the retina dissected in nuclease free ice-cold PBS (137 mM sodium chloride, 2.7 mM potassium chloride, 8 mM sodium phosphate dibasic, 2 mM potassium phosphate monobasic, pH 7.4). The animal procedures were in accordance with the institutional Animal Care and Use Committee and with the standards established by the New York State Department of Health, the USA Public Health Service, the USA Department of Agriculture, and the Association for Assessment and Accreditation of Laboratory Animal Care.

### Cell cultures

Rat-2 fibroblasts (ATCC, Manassas, VA) were maintained in vitro as previously described [21]. Briefly, cells were maintained in Dulbeco’s Modified Eagle Medium (DMEM, Sigma) supplemented with sodium bicarbonate (1.5 g/l; Invitrogen, Carlsbad, CA) and 10% fetal bovine serum (FBS, Invitrogen). Rat-2 cells were seeded at a density of 5.8×10^5^ cells in a T-25 tissue culture flask (Falcon; BD, San Jose, CA) and allowed to adhere overnight. Transfection agent (siPORT-Amine; Ambion, Austin, TX) was diluted (1:40) in Opti-MEM (Invitrogen) and incubated at room temperature for 10 min. Three *CTGF* siRNAs (Ambion) were individually tested for their effect on *CTGF* mRNA and protein. Sequences for these siRNAs are shown in Table 1. Each siRNA (50 mM), diluted in 900 µl of Opti-MEM, was added to the transfection mix and incubated at room temperature for 10 min. The siRNA/transfection agent complexes were added to suspended cells (1.8×10^6^), and the mixture was placed in a T-75 flask in medium containing 10% FBS. siRNA transfected cells were incubated at 37 °C with CO_2_ for 24 h. Then the cells were processed and treated with transforming growth factor (TGF)-β1 (R&D Systems, Minneapolis, MN) as previously described [21]. In brief, cells were incubated with 0.5% FBS DMEM medium containing TGF-β1 (0.1 ng/ml) for 4 or 6 h before harvesting. After TGF-β1 treatment, cells were trypsinized with 0.25% trypsin-EDTA (Trypsin-EDTA; Invitrogen) and pelleted by centrifugation at 200 g for 5 min (International Equipment Company, Chattanooga, TN). Cell pellets were washed in RNase-free ice-cold PBS and centrifuged at 1,300× g (MTX-150; Tomy Tech, Menlo, CA) for 10 min at 4 °C and then used for total RNA isolation or protein extraction.

**Table 1:** Sequences for siRNAs.

**Name of siRNA**	**Sense (5′-3′)**	**Antisense (5′-3′)**
Scrambled siRNA	Sequence undisclosed by company	
*CTGF* siRNA I *	GGAUUACAGUAGCACAUUATT	UAAUGUGCUACUGUAAUCCTT
*CTGF* siRNA II	GGGACACGAACUCAUUUAGTT	CUAAAUGAGUUCGUGUCCCTT
*CTGF* siRNA III	CGAACUCAUUUAGACUAUATT	UAUAGUCUAAAUGAGUUCGTG
Scrambled siRNA*	UAAGGCUAUGAAGAGAUACUU	PGUAUCUCUUCAUAGCCUUAUU

*Denotes siRNA used for intravitreal injections.

### Insulin therapy

A subset of diabetic animals (FBG≥350 mg/dl) was implanted with subcutaneous insulin pellets (LinShin Canada Inc., Toronto, Canada) for 12 weeks, on the first day of hyperglycemia according to the company’s recommendations. FBG was measured 1 week after insulin pellet implantation (day 1 of the experiment for the group treated with insulin), and weekly thereafter. Animals with poor glycemic control received two additional insulin pellets.

### Intravitreal small interfering ribonucleic acid injections

After 12 weeks of hyperglycemia, a subset of rats received a single intravitreal injection of siRNA. Animals were anesthetized with vaporized isoflurane. Eyes were visualized with a dissecting microscope, and the sclera was pierced behind the ora serrata using a borosilicate glass capillary (World Precision Instruments, Sarasota, FL) that was heat pulled to obtain a thin diameter. siRNAs (Table 1) were modified for in vivo studies by Dharmacon (Lafayette, CO), using undisclosed techniques due to proprietary considerations. siRNA was diluted in sterilized, RNase/DNase-free, physiologic PBS for a final concentration of 2.5 µg/µl. siRNA was injected using a sterilized Nanofill syringe attached to a 33G beveled needle (World Precision Instruments) inserted in the opening produced by the capillary needle. One eye was injected with 3 µl (7.5 µg) of CTGF siRNA and the contralateral eye with scrambled siRNA (7.5 µg). Animals were sacrificed at 3 and 10 days post siRNA injection.

To track and visualize siRNA inside the layers of the retina, a Label IT siRNA Tracker Kit (Mirus, Madison, WI) was used to label the scrambled siRNA with Cy3. Briefly, about 10 mg of siRNA (Silencer® Negative Control #2 siRNA; Ambion) was incubated with 5 μl labeling buffer A (Mirus) and 10 μl labeling reagent (Mirus) at 37 °C for 1 h, unreacted Label IT siRNA Tracker Reagent was removed from the labeled siRNA with ethanol precipitation, and labeled siRNA was spun, washed with ethanol 70%, and resuspended in PBS. Purified, labeled siRNA was quantified with spectrophotometry. Five μg (in 2 μl PBS) of labeled siRNA was injected in one eye. The contralateral eye was injected with the same volume of unlabeled siRNA. Eyes were enucleated 3 days later, placed in mounting medium, snap frozen in liquid nitrogen, and cryosectioned, and cells were visualized by staining the nuclei with To-Pro Blue (Invitrogen).

### Immunohistochemistry

Animals were euthanized, and the eyes were immersed in embedding matrix (ThermoFisher, Waltham, MA) and snap frozen in liquid nitrogen. Then 20 µm sections were obtained. The plane of section was parallel to the optic nerve. Cryosections were fixed in ice-cold acetone (10 min), and post-fixed in Zamboni’s fixative for 2 min. Slides were washed in 0.1 M PBS, and sequentially incubated in PBS containing 0.3% hydrogen peroxide for 20 min, in blocking buffer (PBS containing 3% horse serum and 0.01% saponin) for 30 min, and incubated with goat anti-human CTGF antibody (diluted 1:100 in 0.01 M PBS; R&D) at 4 °C overnight. The specificity of this antibody has been previously described [22-26]. The next day, slides were incubated for 1 h with Alexa Fluor 488 conjugated with antigoat immunoglobulin G (diluted 1:200 in 0.01 M PBS; Invitrogen), washed in PBS, fixed in 4% paraformaldehyde for 5 min, and incubated overnight with mouse anti-vimentin antibody (1:500 in 0.01 M PBS). The following day, slides were incubated for 1 h with Alexa-Fluor 594 (1:200 in 0.01 M PBS; Invitrogen) and coverslipped using Vectashield mounting medium (Vector, Burlingame, CA).

### Confocal microscopy

Confocal images were obtained using a Radiance 2000 confocal microscope (Bio-Rad, Hercules, CA) connected to a Zeiss Axioskop microscope (Carl Zeiss, Thornwood, NY). Images of 1240×1240 pixels were processed using Photoshop (Adobe, San Jose, CA). All images were captured using similar settings on the confocal microscope.

### Quantitative real-time polymerase chain reaction

Total RNA was extracted using the RNAeasy Kit (Qiagen, Germantown, MD) as described in Winkler et al. [21]. Briefly, concentration and purity of RNA were determined by spectrophotometry. RNA integrity was verified by electrophoresis in 1.2% denaturing formaldehyde gel (1.2 g agarose, 10 ml 10× FA gel buffer [200 mM 3-[N-morpholino] propanesulfonic acid; MOPS], 50 mM sodium acetate, 10 mM EDTA, pH 7.0) in 100 ml RNase-free water). RNA preparations were treated with recombinant DNase-1 (DNA-free kit; Ambion). One microgram of total RNA was transcribed using SuperScript III reverse transcriptase and oligo(dT)_18_ primer (Invitrogen). Real-time PCR was performed using RT^2^ SYBR Green Mix (SA Biosciences, Frederick, MD) and performed on a StepOnePlus cycler (Applied Biosystems, Carlsbad, CA). The cycling conditions were 95 °C for 10 min, 40 cycles at 95 °C for 15 s, and 60 °C for 1 min. mRNA levels were normalized to an appropriate housekeeping gene, TATA-binding protein (*TBP*) [27] or acidic ribosomal protein P0 (*ARPP_0_*) [28]. Primer sequences for laminin β1, fibronectin 1, and collagen IVα3 were taken from a previous publication [5]. Details of the primers are shown in Table 2. Data was analyzed using the 2^-∆∆C(T)^ method [29]. Each sample was run in triplicate, and each real-time PCR was repeated three times.

**Table 2:** Primer Sequences.

**Gene name**	**5′ Primer**	**3′ Primer**	**Catalog number/reference**
Connective tissue growth factor (*CTGF*)	Purchased from SA Biosciences		PPR46426A
Laminin β1 (*Lam*)	GCGTAAAGCTGCCCAGAACTCTG	TCCTCCTGGCATCTGCTGACTC	[5]
Fibronectin (*FN*)	CAGCCTACGGATGACTCATGC	CAGATAACCGCTCCCATTCCT	[5]
Collagen 4α3 (*Col 4a*)	CCCTTGAGCCCTACGTTAGCA	CCTCAGAGCCTCCACTTGTAAACA	[5]
Acidic ribosomal phosphoprotein P0 (*ARPP0*)	GTCCAACTACTTCCTCAAGATCATCCA	ACATGCGGATCTGCTGCAT	[28]
TATA binding protein (*TBP*)	Purchased from SA Biosciences		PPR47412A

### Western blot analysis

Retinas were homogenized for 5 min in ice-cold lysis buffer (50 mM Tris HCl, pH 7.4, 5 mM EDTA, and 0.02% sodium azide) containing a cocktail of protease inhibitors (Sigma). After homogenization, samples were centrifuged at 28,000× g for 10 min at 4 °C. Protein concentration in cleared lysates was measured using bicinchoninic acid colorimetric assay (BCA kit; Pierce, Rockford, IL). Samples were fractionated on a 15% Tris-HCl sodium dodecyl sulfate–PAGE (SDS–PAGE) Ready Gel (Bio-Rad) for CTGF and VEGF and a 5% Tris-HCl SDS–PAGE Ready Gel (Bio-Rad) for laminin; 30 µg of total protein was loaded into each lane. After electrophoresis, protein samples were electroblotted onto nitrocellulose membrane (Amersham, Pittsburgh, PA), washed in Tris-buffered saline, and incubated overnight at 4 °C with goat anti-CTGF antibody (1:1,000; Santa Cruz, Santa Cruz, CA). Membranes were incubated with horseradish peroxidase–conjugated Donkey anti-goat secondary antibody (1:10,000; Santa Cruz), and bands were visualized using SuperSignal West Pico chemiluminescent detection reagents (Thermo Scientific) and Hyblot-CL Autoradiography Film (Denville, Metuchen, NJ). Membranes were subsequently stripped with Restore Western Blot Stripping (Thermo Scientific), incubated with antibodies to VEGF (1:1,000; Santa Cruz) and to glyceraldehyde 3-phosphate dehydrogenase (GAPDH; 1:1,000; Santa Cruz) for loading control, and processed for immunodetection as described as above. GAPDH was chosen because changes in protein levels have been reported in vivo only after 12 months of hyperglycemia [30]. Different membranes were incubated with anti-laminin sera, a pan-specific antibody that recognizes multiple laminin isoforms (1:1,000; Novus, Littleton, CO). For loading controls, membranes were stained with Ponceau S. Densitometric analysis of the luminescent signal was performed at non-saturating exposures. Bands were scanned with Epson Perfection 2400 and analyzed using Image J software (NIH, Bethesda, MD). Densitometry was performed using proteins that were run on the same gel.

### Statistical analysis

All values are shown as mean±SD. For comparison between two groups, the unpaired Student *t* test (two tail) was used. p≤0.05 was considered significant.

## Results

### Connective tissue growth factor messenger ribonucleic acid and protein increased because of hyperglycemia

**Figure 1:**
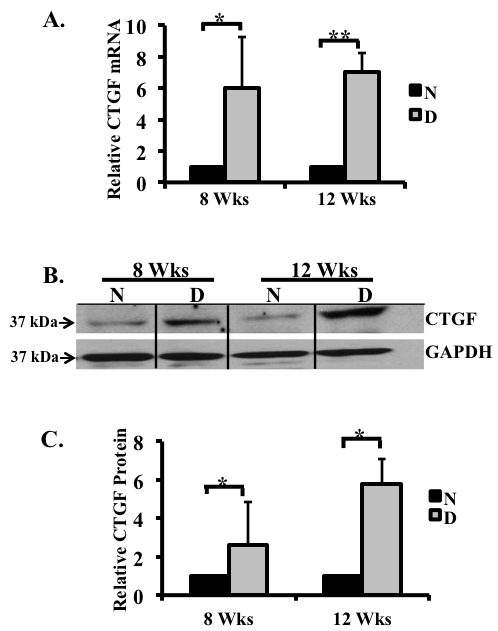
Connective tissue growth factor (CTGF) expression increased in retina of 8 and 12 week diabetic rats. *CTGF* mRNA expression in the retinas of non-diabetic and diabetic rats after 8 and 12 weeks of hyperglycemia were analyzed using real-time PCR and normalized to the housekeeping gene acidic ribosomal phosphoprotein P0 (*ARPP_0_*). **A**: *CTGF* mRNA levels increased six- and sevenfold at both time points. **B**: A representative western blot illustrating CTGF and glyceraldehyde 3-phosphate dehydrogenase (GAPDH) protein expression in the retinas of diabetic rats after 8 and 12 weeks of hyperglycemia. Note the increased CTGF protein levels compared to the non-diabetic controls. **C**: Densitometric analysis of three separate immunoblots indicates a 2.5 and 5.7 fold increase in the CTGF protein after 8 and 12 weeks of hyperglycemia, respectively. (*p<0.05, **p<0.001 diabetic versus non-diabetic) n=non-diabetic; D=diabetic. Proteins ran on a 4%–15% gradient gel. n=3/age.

**Figure 2:**
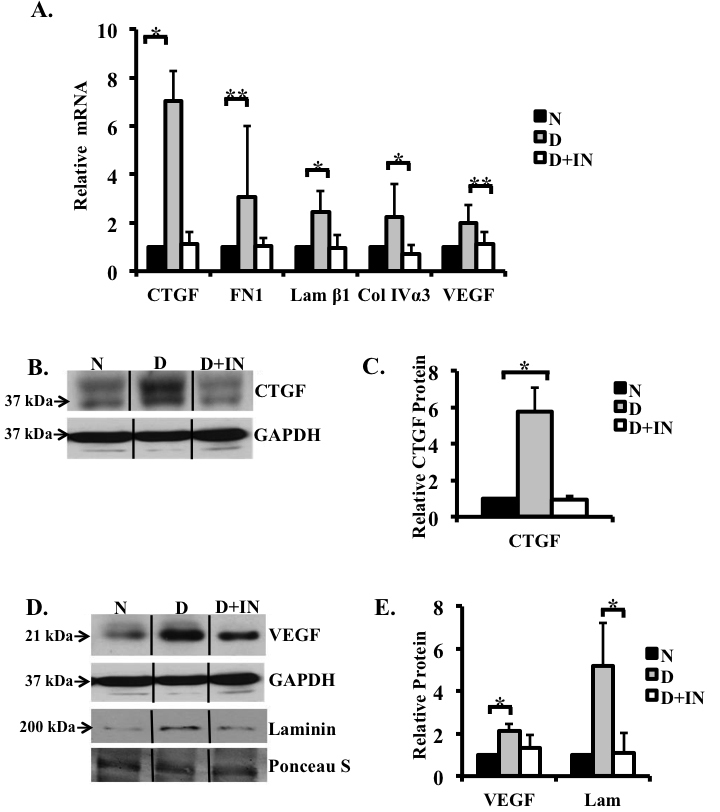
Insulin therapy in diabetic rats abolished hyperglycemia-induced increases in levels of selected genes and proteins. mRNA expression in the retinas of non-diabetic, diabetic, and diabetic rats treated with insulin were analyzed using real-time PCR and normalized to the housekeeping gene acidic ribosomal phosphoprotein P0 (*ARPP_0_*). **A**: Real-time PCR analysis of mRNA extracted from the retina. Note the increase in the levels of connective tissue growth factor (*CTGF*; sevenfold), fibronectin (threefold), laminin β1 (2.5-fold), collagen IVα3 (2.3 fold), and vascular endothelial growth factor (*VEGF*; twofold) mRNAs in the rat retinas after 12 weeks of hyperglycemia and that this increase was prevented with insulin therapy. **B**: A representative immunoblot showing CTGF expression in the retinas of normoglycemic control, diabetic, and insulin-treated rats. Proteins were ran on a 15% SDS–PAGE gel, allowing for better protein separation, hence the double band for CTGF. **C**: Densitometric analysis of three different western blots indicated that the level of CTGF increased following 12 weeks of hyperglycemia, when compared to the non-diabetic controls. In contrast, CTGF expression in the retinas of diabetic rats treated with insulin for 12 weeks remained near the control levels. **D**: A representative western blot for VEGF and laminin in retinal extracts shows an increase in retinal VEGF and laminin in the hyperglycemic state, and this increase was inhibited in the insulin-treated hyperglycemic rats. **E**: Densitometric analysis of three different western blots revealed a 2.1-fold increase in VEGF and a 5.2-fold increase laminin. VEGF and laminin levels were similar in the control and insulin-treated diabetic animals. GAPDH and Ponceau S staining were used as a loading control. n=non-diabetic; D=diabetic; D+IN=insulin treated diabetic (*p<0.05, **p<0.001). n=3/group.

**Figure 3:**
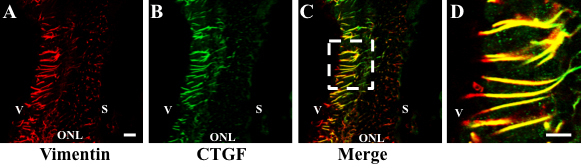
Connective tissue growth factor (CTGF) was detected in vimentin-positive Müller cells in the retina of diabetic rats. **A**: Müller cells labeled with filament protein vimentin throughout the diabetic retina. **B**: CTGF staining in the 12-week diabetic retina is also seen throughout the retina. **C**: Merge photomicrograph for vimentin and CTGF shows that CTGF colocalizes with vimentin in the diabetic retina. Bar=60 um. **D:** High magnification of the area indicated with a dotted rectangle in **C**. Bar=5 um. V=Vitreous, ONL=Outer Nuclear Layer, S=Sclera.

To ascertain whether *CTGF* gene expression increased during hyperglycemia, the level of *CTGF* mRNA and protein in the retina of diabetic and non-diabetic control rats was compared. At 8 and 12 weeks of hyperglycemia, *CTGF* mRNA was sixfold (p<0.05) and sevenfold (p<0.001) higher than in controls, respectively (Figure 1A and Figure 2A). Similarly, CTGF protein increased 2.5 fold (p<0.05) at 8 weeks and 5.7 fold (p<0.05) at 12 weeks of hyperglycemia (Figure 1B,C and Figure 2B,C). These findings are in agreement with previous reports by others [5,12,13]. To ascertain the identity of the cells expressing CTGF, sections of retina were processed for simultaneous visualization of CTGF and vimentin, a marker of Müller cells [31]. This analysis indicated that in the retina of diabetic rats, CTGF is expressed by vimentin-positive Müller cells (Figure 3).

In addition to an increase in *CTGF*, hyperglycemia affected the level of expression of *VEGF* and of possible downstream ECM genes in the retina. Comparison of the retina of 12-week diabetic and normoglycemic rats revealed a significant increase in fibronectin (threefold, p<0.001), laminin β1 (2.5 fold, p<0.05), collagen IVα3 (2.3 fold, p<0.05), and *VEGF* (twofold, p<0.001) mRNAs (Figure 2A). Protein levels of VEGF and laminin also increased significantly (2.1 fold and 5.2 fold, respectively [p<0.05]) in the retina after 12 weeks of hyperglycemia (Figure 2D,E). These results confirm previous observations obtained using a similar model [4,32].

### Insulin therapy regulates the levels of connective tissue growth factor, vascular endothelial growth factor, and extracellular matrix molecules

**Figure 4:**
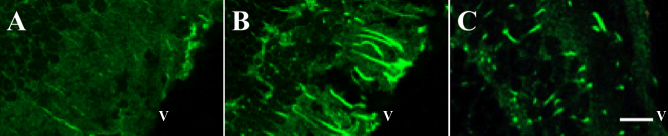
Hyperglycemia induces connective tissue growth factor (CTGF) expression in Müller cells. A representative photomicrograph illustrates retinas processed for visualization of CTGF. The CTGF level is low in the retinas of the control rats (**A**) and increased in the retinas of the diabetic rats after 12 weeks of hyperglycemia (**B)**. Insulin therapy for 12 weeks decreased the staining intensity of CTGF (**C**). All images were captured at the same magnification. V=vitreous side of the retina. Bar=30 um. n=3/group.

To ascertain whether insulin therapy affects the level of expression of *CTGF*, *VEGF*, and possible downstream ECM molecules in the retina, a subset of rats with STZ-induced diabetes received insulin therapy at 7 days post-STZ for 12 weeks. Insulin therapy normalized blood glucose levels and reduced weight loss in diabetic rats (Table 3). The levels of *CTGF*, fibronectin, laminin β1, collagen IVα3, and *VEGF* mRNAs in the retinas of diabetic rats receiving insulin therapy were similar to that of controls (Figure 2A). Similarly, CTGF, VEGF, and laminin proteins were similar in insulin-treated STZ and non-diabetic control (Figure 2B-E). To confirm the effect of insulin on CTGF, the levels of CTGF in the retinas of the control, diabetic, and insulin-treated diabetic rats were compared with immunohistochemistry. The level of CTGF immunostaining was undetectable in control retinas (Figure 4A), increased in diabetic retinas (Figure 4B), and significantly reduced in the retinas of diabetic rats treated with insulin (Figure 4C). In addition, we confirmed previous reports indicating that insulin treatment normalized fibronectin levels in the retinas of diabetic rats (Figure 2A) [33]. These findings indicate that insulin prevented the glucose-dependent increase in the expression of these selected cytokines and ECM genes.

**Table 3:** Average blood glucose and weight of control, diabetic and insulin treated animals.

**Parameter**	**Control (non-diabetic) n=6**	**Diabetic (untreated) n=12**	**Insulin treated diabetic n=8**
Blood glucose (mg/dl)	117±13.8	405±68	135±89
Weight (g)	729±52	335±71	524±60

### Effect of connective tissue growth factor small interfering ribonucleic acid on the expression of connective tissue growth factor and possible downstream targets

**Figure 5:**
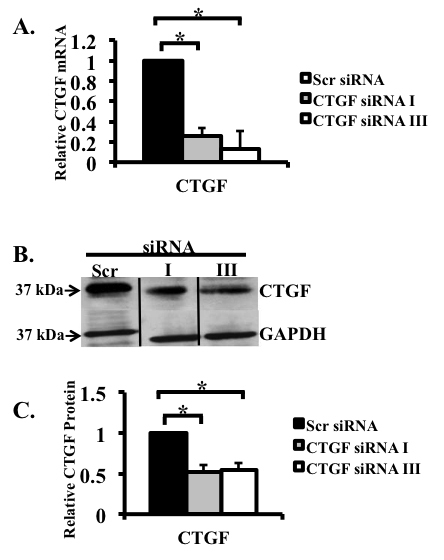
siRNA decreased transforming growth factor-β1 (TGF-β)-induced connective tissue growth factor (*CTGF*) in Rat-2 fibroblasts. *CTGF* mRNA expression in Rat-2 cells treated with TGF-β and siRNA were analyzed using real-time PCR and normalized to the housekeeping gene acidic ribosomal phosphoprotein P0 (*ARPP_0_*). **A**: The effect of two separate *CTGF* siRNAs (I and III) and a control scrambled siRNA on TGF β-induced *CTGF* expression in Rat-2 fibroblasts was tested. Real-time PCR showed that siRNA I and siRNA III decreased *CTGF* mRNA by 75% and 86%, respectively. **B**: Arepresentative western blot documenting that TGF-β induced CTGF protein decreased by siRNA I and III. Proteins were run on the same gel, but not in adjacent lanes. **C**: Densitometric analysis summarizing the results of three separate western blots shows that siRNA I and siRNA III decreased CTGF protein by 49% and 46%, respectively. (*p<0.05). n=3/group.

To further analyze the contribution of *CTGF* in regulating ECM molecules, we developed a siRNA-approach to inhibit *CTGF*. We tested if inhibition of *CTGF* by siRNA affected the expression of laminin β1, fibronectin 1, collagen IVα3, and *VEGF*. In addition, we also tested the effect of the treatment on glial fibrillary acidic protein (GFAP), an intermediate filament protein that increases in the retina during DR [34,35],

We first used an in vitro system to determine the specificity of three siRNAs in downregulating *CTGF* levels in a rat cell line. Since we previously showed that TGF-β induces a twofold increase in *CTGF* mRNA and a significant increase in the CTGF protein in Rat-2 fibroblasts [21], we tested whether the induction of *CTGF* was inhibited by any of the three *CTGF* siRNAs (siRNA I–III) alone or in combination. Previous studies showed that CTGF protein levels in Rat-2 cells were undetectable in the absence of TGF-β [21]. Real-time PCR showed that siRNA I and siRNA III decreased TGF-β induced *CTGF* mRNA by 75% and 86% (p<0.05), respectively (Figure 5A). In contrast, siRNA II had no effect on reducing *CTGF* mRNA (data not shown). Western blot analysis using siRNA I and siRNA III revealed that CTGF protein decreased by 49% and 46%, respectively (Figure 5B,C). The combination of siRNA I and III did not result in further inhibition of *CTGF* (data not shown). siRNA I was chosen for the in vivo experiments because it gave the greatest decrease in CTGF protein.

**Figure 6:**
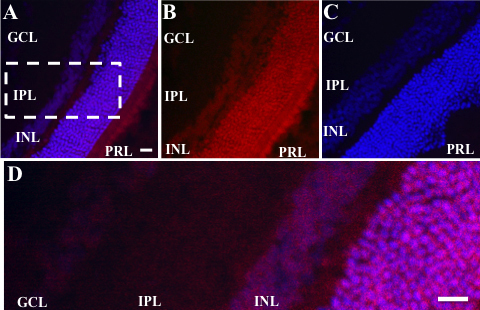
Intravitreal injection of labeled siRNA penetrates the retina. **A**: Confocal microscopy revealed that the scrambled siRNA covalently bound to Cy3 (Red) was distributed throughout the layers of the retina when injected into the eyes of control rats. The label was distributed throughout the retina from the ganglion cell layer (GCL) to the photoreceptor layer (PRL). Nuclei were stained with To-Pro Blue. **B**: A photomicrograph illustrates panel **A** with To-Pro Blue removed. Note the distribution of Cy3 labeled siRNA throughout the layers of the retina. **C**: In contrast, the retinas of rats injected with unlabeled scrambled siRNA did not contain labeled particles, indicating that the label was not due to autofluorescence or artifacts from the injection. Bar=30 um. **D:** Illustrates higher magnification of inset depicted with dotted rectangle in **A**. Bar=15 um. IPL=inner plexiform layer, INL=inner nuclear layer. n=3/group.

Then we sought to determine whether the siRNA reached all layers of the retina. Animals were given a single intravitreal injection of Cy3-labeled scrambled siRNA, and the eyes were harvested 3 days after the injection, sectioned, and viewed using confocal microscopy. Labeled siRNA was distributed throughout the retina from the ganglion cell to the photoreceptor layers (Figure 6A,B,D). The contralateral eyes were injected with unlabeled siRNA to test whether the light emissions observed in the eyes injected with labeled siRNA was due to autofluorescence produced by the siRNA. Retinas of rats that received unlabeled siRNA did not show any detectable fluorescence (Figure 6C). The distribution of labeled siRNA throughout the retina confirms previous reports by Shen et al. [36].

**Figure 7:**
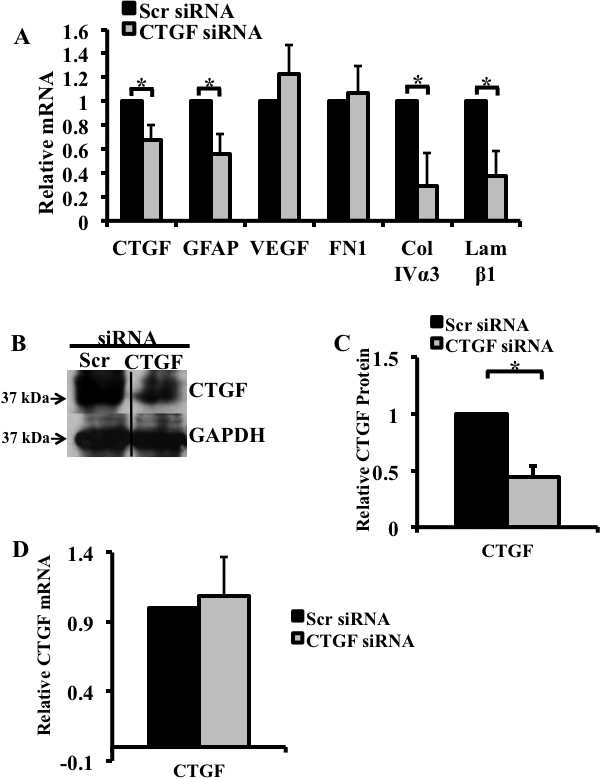
siRNA treatment significantly decreased hyperglycemia-induced increase in connective tissue growth factor (CTGF) mRNA and protein, glial fibrillary acidic protein (GFAP), collagen IVα3 and laminin-β1 gene expression. mRNA expression for *CTGF* and selected genes in the retinas of the diabetic rats after 12 weeks of hyperglycemia were analyzed using real-time PCR and normalized to the TATA-binding protein (*TBP*). **A**: Real-time PCR revealed that 3 days post intravitreal injection, *CTGF* siRNA induced a decrease in *CTGF* (33%), *GFAP* (44%), collagen IVα3 (71%), and laminin β1 (63%) mRNAs. In contrast, *CTGF* siRNA did not affect the level of fibronectin or vascular endothelial growth factor (*VEGF*). **B**: Immunoblot analysis of CTGF levels in the retinas following a single intravitreal injection of *CTGF* siRNA or scrambled siRNA into left and right eye, respectively. The concentration of the CTGF protein is lower in eyes injected with *CTGF* siRNA. **C**: Densitometric analysis of three independent experiments revealed a 54% decrease in CTGF protein in retinas injected with *CTGF* siRNA compared to retinas injected with a scrambled (non-specific) siRNA. Glyceraldehyde 3-phosphate dehydrogenase (GAPDH) was used as loading control. **D**: Real-time PCR showed that *CTGF* siRNA had no effect on *CTGF* expression 10 days after injection. (*p<0.05). n=5/group.

To determine whether the siRNA treatment had a beneficial effect on the retinas of diabetic rats, 12-week hyperglycemic rats were given a single intravitreal injection of *CTGF* siRNA in one eye (the experimental retina), while the contralateral eye was injected with scrambled siRNA (the control retina). Three days later, the retinas were dissected and processed for mRNA and protein analysis. Administering *CTGF* siRNA induced a decrease in *CTGF* (33%, p<0.05), collagen IVα3 (71%, p<0.05), and laminin β1 (63%, p<0.05) mRNA levels (Figure 7A). Levels of *GFAP* mRNA decreased by 44% (p<0.05) in the *CTGF* siRNA–treated retina (Figure 7A). Importantly, the level of CTGF protein was 54% lower (p<0.05) in the experimental retinas compared to the control retinas (Figure 7B-C). In contrast, administering *CTGF* siRNA did not affect VEGF or fibronectin levels, which remained similar in the experimental and control retinas (Figure 7A). The effect of the siRNA treatment decreased with time since the *CTGF* mRNA levels were similar in the experimental and control retinas 10 days after *CTGF* siRNA was injected (Figure 7D).

## Discussion

Although insulin therapy aids in preventing DR, since insulin stabilizes endothelial barrier function [37] and decreases microvascular leakage [38], the role of insulin in regulating ECM molecules is not well defined. In this study, our first goal was to determine whether CTGF, a profibrotic molecule induced by high glucose [5,13], was downregulated with insulin therapy and whether this decrease also affected extracellular matrix components proposed to be controlled by CTGF [19]. We found that the elevated levels of *CTGF* mRNA and protein induced by hyperglycemia were prevented by exogenous insulin treatment. Moreover, insulin therapy also inhibited the upregulation of several components of the extracellular matrix such as laminin β1, collagen IVα3, and fibronectin, and of *GFAP* and *VEGF*.

Importantly, we determined that Müller cells in the diabetic retina immunostained with a specific antibody to CTGF. Although CTGF is a secreted protein, it is not surprising that it is localized within the cytoplasm of Müller cells. Other secreted proteins, such as hormones, are also localized to the cytoplasm of the cells that synthesize them. In contrast to our findings, previous immunohistochemical studies reported the expression of *CTGF* by cells of the ganglion cell and inner plexiform layers in rodents [5,15] and in microglial cells and pericytes in humans [12]. The discrepancy between our results and that of others could be due to the different histological techniques employed in the analysis and/or to the specificity of the antibodies used. In situ hybridization studies reported the expression of *CTGF* mRNA by structures located in the ganglion cell layer [13] and proposed that it was expressed by the end feet of Müller cells [13]. Although in situ hybridization identifies the site of mRNA expression, identifying the cell type that is labeled is often difficult. For that reason, we used immunohistochemistry to visualize CTGF. Our results are in agreement with the cell type proposed by that group to synthesize CTGF. The antibody did not stain CTGF bound to the cell membrane of cells that respond to the action of this cytokine. Although a specific CTGF receptor has not yet been identified, CTGF appears to perform many of its functions through integrins, heparin sulfate–containing proteoglycans, and the low-density lipoprotein receptor–related protein [39]. Although CTGF probably binds to any or all of these receptors, its levels at the cell membrane are probably below the sensitivity of the techniques used.

Müller cells become activated by high glucose levels and upregulate the expression of *GFAP*, and of *VEGF* and other angiogenic cytokines [35,40]. The localization of CTGF to Müller cells has important clinical applications since these cells participate in maintaining the homeostasis of the retinal extracellular milieu and protect neurons via a release of neurotrophic factors; disturbance of these cells could lead to retinal dysfunction [40].

Although insulin therapy downregulated the level of expression of *CTGF* and ECM molecules, oscillations in blood glucose occur even in the presence of tight glycemic control [41], and these changes could affect retinal function even in well controlled patients [42]. To circumvent possible harmful effects of blood glucose oscillations, we sought to develop a siRNA approach for short-term inhibition of *CTGF*. Our goal was to determine whether a reduction in *CTGF* expression in diabetic rats affected the activity of genes regulating the synthesis of selected ECM molecules. We found that the siRNA treatment specifically decreased *CTGF* expression in vitro and in vivo. In vivo, siRNA molecules labeled with Cy3 were found in all layers of the retina. This observation suggests that the injected molecule reaches all retinal cells but affects only the level of *CTGF* expression by Müller cells. Since other retinal cell types do not express *CTGF*, their gene expression is not likely to be affected due to the exquisite specificity of the siRNA approach.

We demonstrated that siRNA treatment decreased *CTGF* mRNA and protein. This effect is specific, because the treatment downregulated the 36–38 kDa form of CTGF, the primary form of the cytokine [43]. In contrast to our finding, a recent study using a similar approach reported a decrease in a 55 kDa protein probed with a CTGF antibody and no change in the 36–38 kDa forms [44]. The identity of the 55 kDa protein is unknown. Moreover, the specificity of the siRNA treatment in Yang et al.’s report is unclear since the synthetic oligonucleotides used in that report also decreased VEGF [44]. In contrast, in our study the *CTGF* siRNA did not affect *VEGF* mRNA levels, supporting multiple reports indicating that *VEGF* is upstream of *CTGF* [14,15,45].

Our results reveal that the *CTGF*-specific siRNA resulted in a decrease in laminin β1 and collagen IVα3 mRNA levels, indicating that these two genes are CTGF targets. In contrast, inhibition of *CTGF* did not change fibronectin mRNA levels. This result was unexpected because studies in other tissues reported that CTGF regulates fibronectin expression [19,46]. This discrepancy could be due to differences in the half-life of fibronectin mRNA in different cell types. We also found that *CTGF* siRNA reduced *GFAP* expression, indicating that CTGF regulates the level of expression not only of extracellular molecules but also genes coding for intracellular proteins. We did not examine the protein levels of ECM molecules following the siRNA treatment. The short duration of action of the siRNA and the relatively long half-life of extracellular matrix proteins [47] suggest that, in our studies, the siRNA treatment did not affect protein levels.

Although the siRNA experiments support a role for *CTGF* in regulating ECM molecules, the effect of the injected siRNA in diabetic rats lasted for only 3 days since the experimental and contralateral control retinas expressed similar levels of *CTGF* mRNA 10 days after the injection. Other studies also reported a short duration of siRNA treatments [36,48]. Therefore, although our findings support the use of an siRNA approach to complement insulin therapy, future studies should seek to circumvent its limitations. One limitation of siRNA treatment is the ability of siRNA to nonspecifically bind to toll-like receptor 3, resulting in an off-target antiangiogenic response [49]. In addition, the approach requires the development of chemically modified siRNA molecules that will stabilize and extend the duration of the treatment when combined with appropriate transfection reagents. In conclusion, our results clearly demonstrate that CTGF regulates the level of expression of ECM molecules as well as GFAP, a specific glial cell filament.
